# Characterizing regional radon-in-air levels in rocks of the Canary Islands (Spain): new data and results

**DOI:** 10.1007/s10653-022-01202-2

**Published:** 2022-01-20

**Authors:** Jose A. Rodríguez-Losada, Antonio Eff-Darwich, Luis E. Hernández Gutiérrez, Ronaldo Viñas Pérez

**Affiliations:** 1 Departamento de Biología Animal, Edafología Y Geología, Universidad de La Laguna, Av. Astrofísico Francisco Sánchez S/N, 38206 La Laguna, Tenerife, Spain; 2 Instituto Volcanológico de Canarias” (INVOLCAN), Tenerife, Canary Islands, Spain; 3 Government of Canary Islands, Ministry of Education and Universities, Santa Cruz de Tenerife, Spain

**Keywords:** Radon monitoring, Canary Archipelago, Volcanic, Boreholes, Wells

## Abstract

In this work, a regional-scale strategy to characterize the radon activity levels in the Canary Islands (Spain) is described. The main objectives of this strategy consisted of (1) studying the likely relationship between radon concentration and lithology of the rock matrix through the lithological data of 247 samples from volcanic rocks of the Canary Islands and (2) implementing a series of monitoring sites in the form of boreholes and wells to study the evolution of radon-in-air activity on a daily to yearly timescale.

## Introduction

The presence of natural radioactivity is partly due to the presence of primordial radionuclides contained within the Earth crust, such as ^40^ K, ^238^U, ^232^Th and the products of their decay series. In this sense, the levels of gamma radiation (external radiation) in naturally occurring radioactive materials (NORMs) depend upon their contents of thorium, uranium, radium and potassium, whereas the internal exposure occurs due to the inhalation of radon and associated progeny, a decay product of ^226^Ra (UNSCEAR, 2000).

The characterization of radon emissions in the Canarian Archipelago started almost 40 years ago in a pioneering work carried out by Ortiz and Valentín (1983). The research continued in two different areas, namely geology (radon as a monitoring tool of seismo-volcanic activity) and human health (the link between radon concentration and lung cancer). The potential of measuring radon flux as a proxy of changes in geological activity has been extensively studied in the Canary Islands (i.e., Padilla et al. (2013), Viñas et al., (2007), Pérez et al. (2007), Martin-Luis et al. (2002) among others), as well as the evolution of radon-in-air activity over long periods of time (Botha et al., 2018; Moreno et al., 2016), whereas Fernández-Aldecoa et al. (1992), Sainz et al. (2007), Quindós Poncela et al. (2004) and Ruano-Raviña et al. (2014), among others, have worked on the relation between radon concentration and the likelihood of being affected by lung cancer. In previous works, numerical models (Eff-Darwich et al., 2002, 2008) and time-series analysis techniques (Viñas et al., 2007) were also implemented to characterize radon transport and release, whereas intercomparison exercises between different radon detectors (Viñas et al., 2004) were also carried out.

These previous works confirmed that different geological parameters play a significant role in the dynamics of radon. In this sense, this work explores the relationship between geochemical and radiological properties of rock samples, whereas the vertical underground transport of radon is studied in a series of boreholes and wells.

## Geological context

The Canarian Archipelago is comprised of seven major islands: Fuerteventura, Lanzarote, Gran Canaria, Tenerife, La Gomera, La Palma and El Hierro, as well as four smaller islets: La Graciosa, Montaña Clara, Alegranza and Lobos Island. The easternmost islands, Lanzarote and Fuerteventura, are approximately 100 km away from the Northwestern African coast. All of them have been active in the last million years except for La Gomera island. The complete volcano-stratigraphic succession can be summarized into three large units: (l) basal complexes (or pre-shield stage), composed of ocean bottom sediments intruded by sheeted dike swarms, and by plutonic bodies, mainly gabbros, syenites and other minor alkaline intrusions; (2) shield volcanoes composed of dominant basaltic thin lava flows successions known as the Old Basaltic Series; and (3) post-shield volcanism. The successive stages are commonly separated from each other by time gaps that could last millions of years. There is a wide variety of lithologies which include alkali basalts, basanites, tholeiites, tephrites, trachybasalts, trachytes, phonolites and minor rhyolites, among others. Most basalts are of alkaline character. Trachytes and phonolites are very common, being related to the formation of large calderas like those outcropping in Gran Canaria and Tenerife. The islands are built on large, tilted blocks differentially uplifted from the sea floor, as might be deduced from sedimentary or volcanic submarine materials cropping out at different heights above sea level. The degrees of elevation are variable depending on each island (Fuster et al., 1968; Robertson & Stillman, 1979; Staudigel & Schmincke, 1984). The archipelago has a long record of volcanic activity that has been extensively studied over the last three decades by different authors (Ancochea et al., 1990; Cantagrel et al., 1993; Coello et al., 1992; Le Bas et al., 1986; Schmincke, 1979; Staudigel et al., 1986 among others). An interesting chronological feature of the Canary Archipelago is that every comparable unit (basal complexes, shield volcanoes stage and post-shield series) is older in the eastern islands than in the western ones. Thus, the age of the basal complex outcropping in La Palma Island (3–4 Ma) represents only a small fraction of the age of the Fuerteventura basal complex.

## Materials and methods

This work aims to implement a strategy to understand the dynamics of radon at a regional scale, in this case, the Canary Islands. Different methodologies have been designed for this purpose, namely geochemical and lithological characterization, analysis of radon exhalation rates, quantification of radiological hazards and monitoring radon concentration in boreholes and wells.

### Sample collection and geochemical characterization

**Fig. 1:**
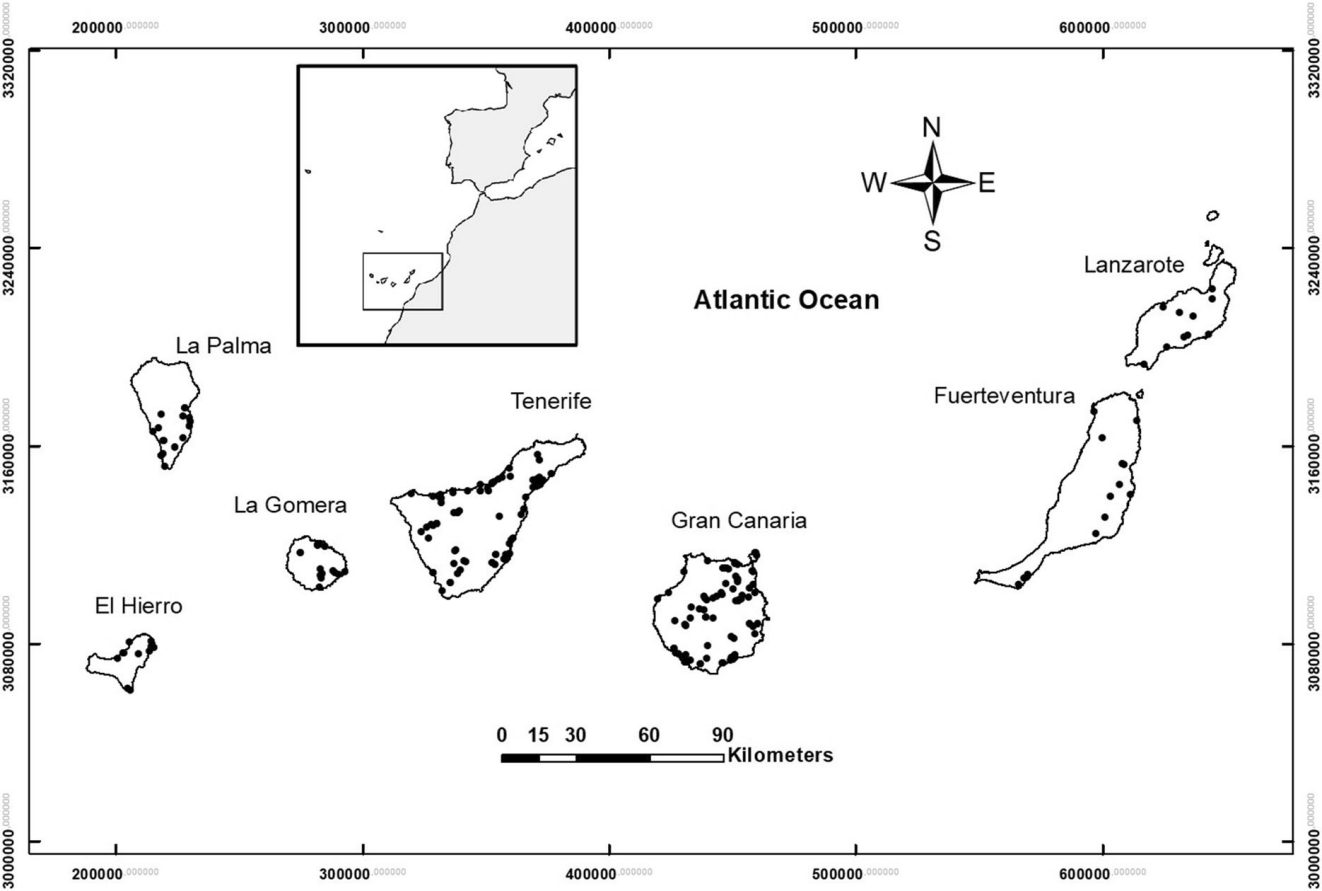
Map of sampling in the different islands of the Canary Archipelago. UTM coordinate system (wgs84)

A collection of 247 rock samples (Fig. 1), square blocks weighing approximately 50 kg, were sampled from all the Canary Islands, to carry out a complete petrographic and geochemical characterization. Rock thin sections were prepared for the petrographic study of each sample in the Geology Laboratory of the University of La Laguna. Geochemical characterization of major and trace elements was carried out in Activation Laboratories Ltd. “ACTLABS” (Canada). Major elements were analyzed by melting and inductively coupled plasma (FUS-ICP) with data given in weight percent and detection limits between 0.01 and 0.001% depending on the element. Trace elements were analyzed by melting and mass spectrometry (FUS-MS) with concentration data given in ppm and lower limits of detection ranging from 30 to 0.002 ppm depending on the analyzed element. Although the number of geochemical species that were analyzed is large, it was decided the use of the silica (SiO_2_) content as the parameter to define the lithological type that the rock sample belongs to, since it gives a good estimate of the differentiation degree of the magmas. Additionally, six selected samples were analyzed by gamma spectrometry in the Environmental Radioactivity Laboratory at the University of Cantabria (Spain) for determining the activity of the radioisotopes ^226^Ra ^238^U, ^236^Th and ^40^K.

The geochemical characterization of all rock samples did not include the determination of the concentration of radium (Ra). The latter was estimated based on the activity concentrations of ^226^Ra, ^232^Th, ^40^K and ^238^U obtained by gamma spectrometry for the six selected samples aforementioned (University of Cantabria). Let’s assign ^232^Th^*^, ^40^K^*^ and ^238^U^*^ as the specific activities (Bq/kg) calculated from the measured elemental concentrations (ppm), obtained from the geochemical analyses of the rock samples (ActLabs, Canada), using the conversion factors of IAEA (IAEA, 2003), recalling that 1 ppm U = 12.35 Bq/kg ^238^U, 1 ppm Th = 4.06 Bq/kg ^232^Th and 1% K = 313 Bq/kg ^40^ K (Table 1).

**Table 1:** Specific activities of radioisotopes obtained from gamma spectrometry (University of Cantabria) and from the elemental concentrations (ppm) of the geochemical analysis (ActLabs)

	^238^U (^234^Th)	^238^U*	^232^Th (^228^Ac)	^232^Th*	^40^ K	^40^ K*	^226^Ra (^214^Bi)
FV-3 (Bq/Kg)	86.6 ± 12.1	78.8	118.4 ± 7.5	108.8	1308 ± 53	1491.2	62.8 ± 2.0
TF-43 (Bq/Kg)	–	47.6	79.4 ± 6.5	64.1	820 ± 34	761.2	42.0 ± 1.5
LP-21 (Bq/Kg)	–	20.7	26.8 ± 2.2	22.3	347 ± 15	345.5	33.2 ± 1.2
FV-11 (Bq/Kg)	–	130.9	134.4 ± 8.2	129.1	1252 ± 50	1353.5	83.2 ± 2.4
LP-19 (Bq/Kg)	–	82.2	97.4 ± 7.9	93.3	910 ± 37	919.6	82.3 ± 2.3
TF-145(Bq/Kg)	–	23.7	24.9 ± 2.1	32.2	524 ± 22	639.1	21.3 ± 0.9

See text for details

From the six samples presented in Table 1, a simple regression analysis was performed in an attempt to find a correlation between the ^226^Ra obtained by gamma spectrometry with respect to ^232^Th^*^ and ^238^U^*^ (Bq/kg) obtained by the conversion of ppm to Bq/kg. The following results were found:

1 $$^{226} {\text{Ra}}\left( {{\text{Bq}}/{\text{kg}}} \right) = 0.5858^{232} {\text{Th}}^{*} \left( {{\text{Bq}}/{\text{kg}}} \right) + 8.9138\left( {R^{2} = 0.84} \right) $$

and

2 $$^{226} {\text{Ra}}\left( {{\text{Bq}}/{\text{kg}}} \right) = 0.6001^{238} {\text{U}}^{*} \left( {{\text{Bq}}/{\text{kg}}} \right) + 14.751\left( {R^{2} = 0.87} \right) $$

The average value from Eqs. (1) and (2) corresponds to the assigned value of ^226^Ra activity, whereas the uncertainties correspond to the differences between both estimates of ^226^Ra. In summary, the analysis of the six samples shown in Table 1 results in Eqs. (1) and (2), from where it is possible to calculate an estimate for ^226^Ra for all rock samples, using the concentrations in ppm of U and Th.

### Radon exhalation rates in rock samples

**Fig. 2:**
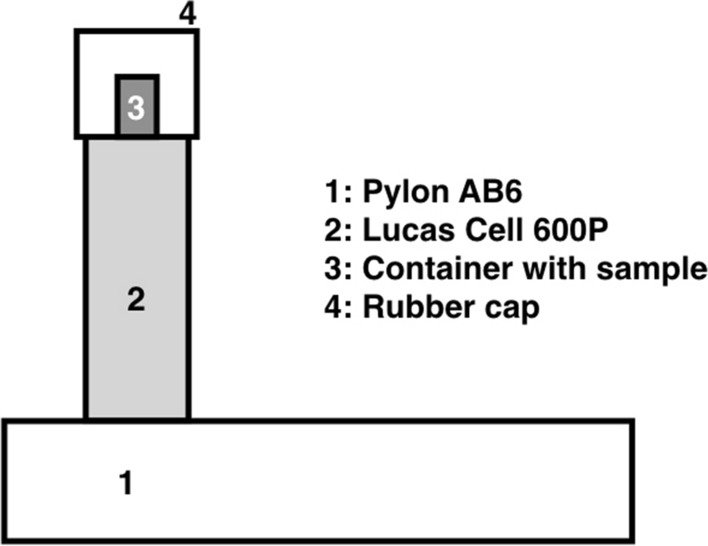
Sketch of the instrumental set up to calculate radon exhalation rates from rock samples. The close chamber is composed of the Lucas cell (2) and the rubber cap (4). The sample is contained in (3). The Lucas cell is attached to the Pylon monitor (1)

**Fig. 3:**
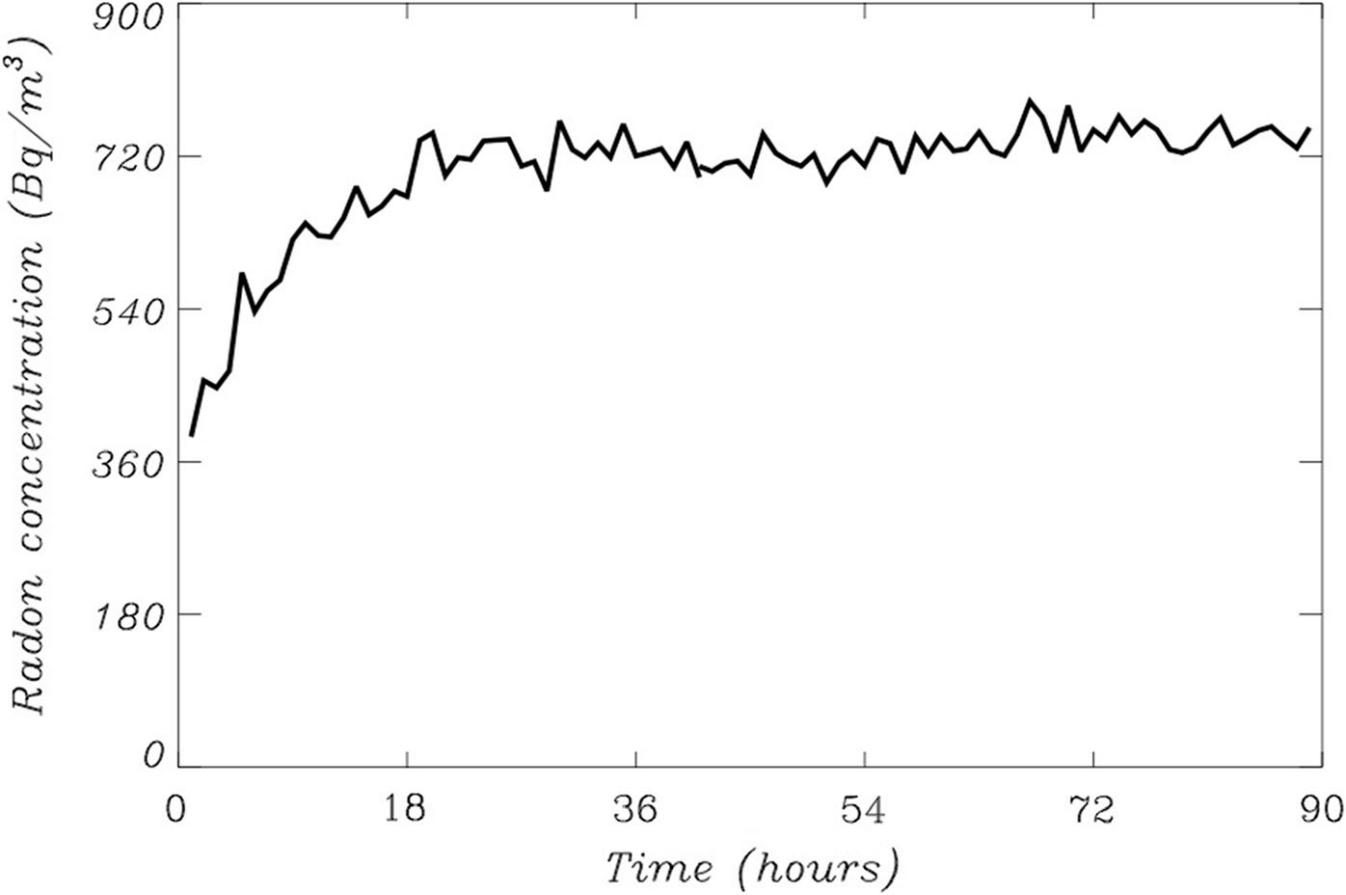
Distribution of radon concentration as a function of time within the close chamber

The release of radon from rocks (or other materials) into the atmosphere is controlled by the exhalation rate (Khan et al., 1992). Two different techniques were used to measure the exhalation rate. The first one, not detailed here, uses a silicon alpha detector AS/BS 1200 V/E from Sarad Instruments placed inside a canister close to the sample and with pumped air flow (roughly 0.6 L per minute) continuously injected into the container. The second one, detailed in this work, is a variation of the ‘close chamber’ technique (Fig. 2). A plastic can, containing the rock samples that have been fine-grained powdered, is placed on top of a Lucas Cell (model 600P) attached to a Pylon AB6 monitor (https://pylonelectronics-radon.com/monitors/). The ‘close chamber’ consists in the Lucas cell and a rubber cap tightly attached to it. The chamber is left sealed for 3 to 4 days until a constant radon concentration is reached (Fig. 3). Final results exhibited no relevant differences between both procedures.

The radon exhalation rate *E*_*A*_ (mBq m^−2^ h^−1^) was obtained by using Eq. 1 (Khan et al., 1992)

3 $${E}_{A}=\frac{CV{\lambda }_{e}}{A\left[T+\frac{{e}^{-{\lambda }_{e}T}-1}{{\lambda }_{e}}\right]}$$

where *E*_*A*_ is the surface exhalation rate, *C* is the integrated radon exposure (Bq/m^3^ h), *V* is the volume of air in the container (m^3^), *T* is the exposure time (*h*) and *λ*_*e*_ is the effective decay constant (h^−1^) that takes into account the decay rate for radon, the leakage of the container and the back diffusion coefficient (Kumar & Chauhan, 2015). The parameter *A* represents the open surface area of the can where the sample is placed (m^2^). In this case, *A* = 3 × 10^–3^ m^2^ and *V* = 4.2 × 10^–4^ m^3^, whereas the volume of the rocks samples within the plastic container was set to 3 × 10^–5^ m^3^. The radon exhalation rate is calculated after fitting Eq. (3) to the temporal distribution of radon concentration measured in the close chamber, like that presented in Fig. 3.

### Radiation hazard indices

Radiological hazards were calculated from uranium, radium, thorium and potassium activity concentrations, in terms of two hazard indexes (Mujahid el al., 2008) and the absorbed dose rate. The external hazard index (*H*_*e*_) is defined as:

4 $$ H_{(e)} = {\text{C}}_{{{\text{Ra}}}} /370 + {\text{C}}_{{{\text{Th}}}} /259 + {\text{C}}_{{\text{k}}} /4810 $$

where *C*_Ra_, *C*_Th_ and *C*_k_ are the specific activities (Bq/kg) of radium, thorium and potassium. The internal exposure to carcinogenic radon and its short-lived progeny is quantified by the internal hazard index *H*_(*i*)_:

5 $$ {\text{H}}_{(i)} = C_{{{\text{Ra}}}} /185 + C_{{{\text{Th}}}} /259 + C_{k} /4810 $$

The standard safety criterion requires that in both cases *H*_(*e*)_ < 1 and *H*_(*i*)_ < 1. The absorbed net dose rate was calculated following the equation proposed in UNSCEAR (1988):

6 $$ D = 0.427C_{{\text{u}}} + 0.662C_{{{\text{Th}}}} + 0.0432C_{{\text{k}}} $$

with *D* being the absorbed dose rate in nGy h^−1^, whereas C_u_, C_Th_ and C_k_ correspond to the specific activities of ^238^U, ^232^Th and ^40^ K expressed in Bq/kg.

### Radon in boreholes and wells

Radon-in-air measurements in four boreholes and two wells (Table 2) were carried out with a Radon Scout from Sarad Environmental Instruments registering radon activity and temperature at intervals of 3 h. These instruments are powered by two A13 batteries of 1.5 V with a duration, under continuous measurement of about 2 to 3 months depending on the interval of data collection. The instrument has a measurement range from 0 to 10 MBq/m^3^ and a sensitivity of 100 Bq/m^3^ with 17% statistical error (1*σ*) at 3 h interval.

**Table 2:** Description of the boreholes and wells used to monitor radon concentration

Site	Type of site	Altitude (m amsl)	Island	UTM coordinate X	UTM coordinate Y
El Portillo	45 m borehole	2135	Tenerife	346,175,62	3,130,770,86
Tejeguate	Well	176	El Hierro	201,982,56	3,074,102,75
Llanillos	Well	175	El Hierro	200,454,35	3,073,724,25
LOP_1m	1 m borehole	544	Tenerife	369,586,81	3,146,438,75
LOP_20m	20 m borehole	544	Tenerife	369,584,09	3,146,440,25
Izaña observatory	30 m borehole	2344	Tenerife	352,751,47	3,131,939,88

In wells, the instruments were placed at a depth of 50 m below the surface, well above the level of the aquifer (approximately 150 m deep). The Radon Scouts were enclosed in a perforated PVC container and suspended from a wire attached to the external frame of the well. This setup protected the instrument from the rain and falling objects.

In boreholes, the Radon Scout was placed in a PVC cylinder that was perforated at the bottom for air circulation. The instrument was hanging from a wire attached to the external frame of the borehole. Unlike the wells, the boreholes were hermetically sealed to prevent direct air circulation with the external atmosphere.

## Geochemical and radiological relationships

In previous studies, the relationships between lithology and radiology in rocks and soils of the eastern Canary Islands were already analyzed (Arnedo et al., 2017). In this work, the relationships between geochemical and radiological parameters of the main lithological types of the Canary Islands are analyzed. At present, 247 samples of volcanic rocks have been studied, of which 37 were selected to determine the connection between lithology and radon exhalation rate.

**Fig. 4:**
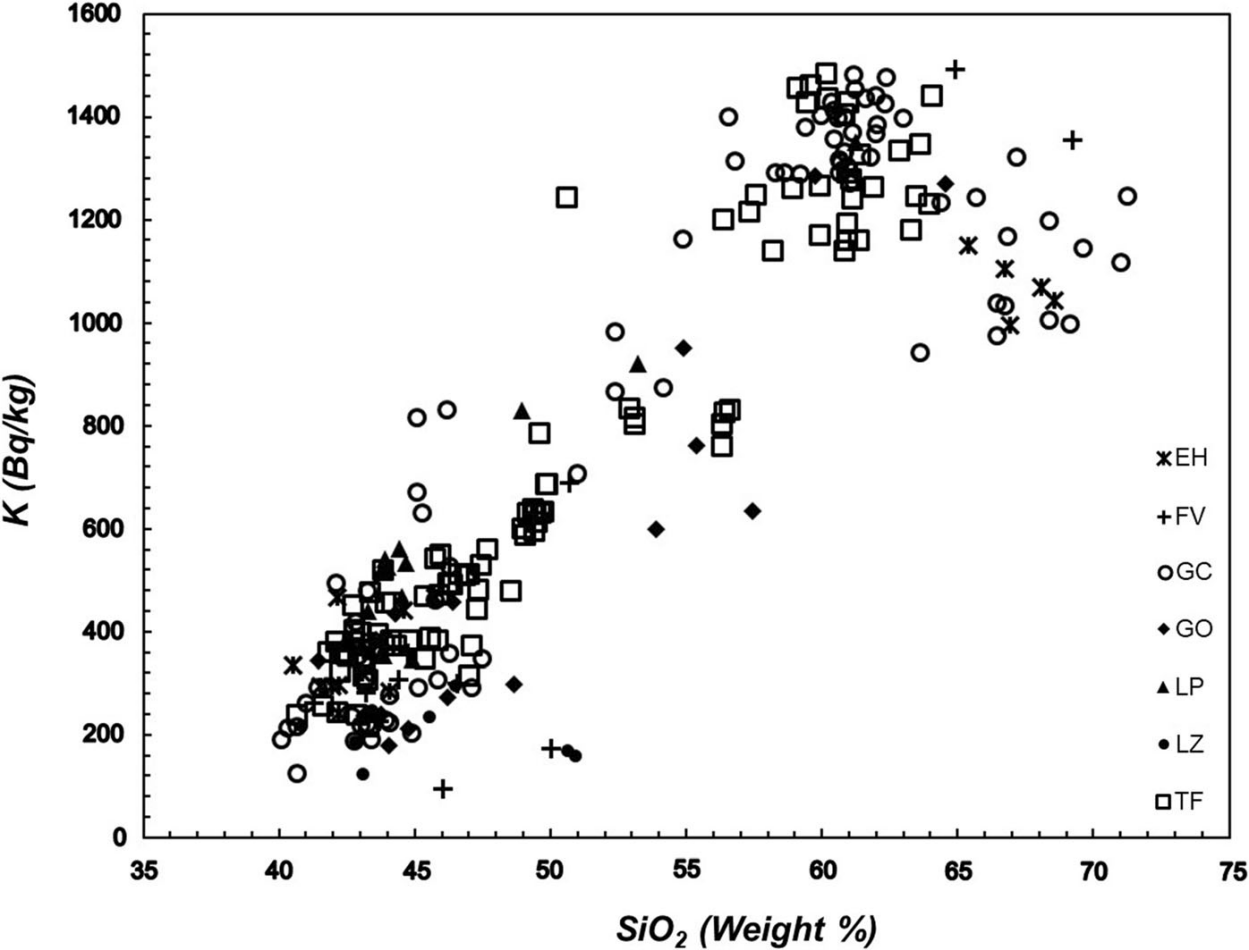
Specific activity of potassium versus silica content. Detection limits: SiO_2_ (0.01%), K (3.1 Bq/kg). Symbols explanation: El Hierro (EH); Fuerteventura (FV); Gran Canaria (GC); La Gomera (GO); La Palma (LP); Lanzarote (LZ); Tenerife (TF)

**Fig. 5:**
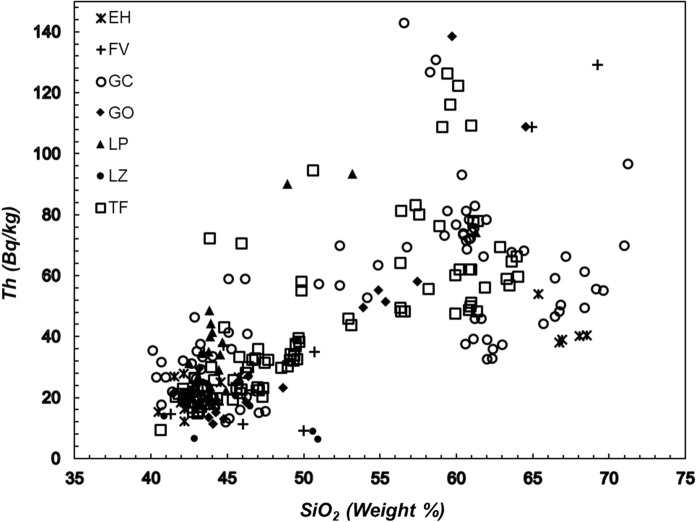
Specific activity of thorium versus silica content. Detection limits: SiO_2_ (0.01%), Th (0.20 Bq/kg). Symbols explanation: El Hierro (EH); Fuerteventura (FV); Gran Canaria (GC); La Gomera (GO); La Palma (LP); Lanzarote (LZ); Tenerife (TF)

**Fig. 6:**
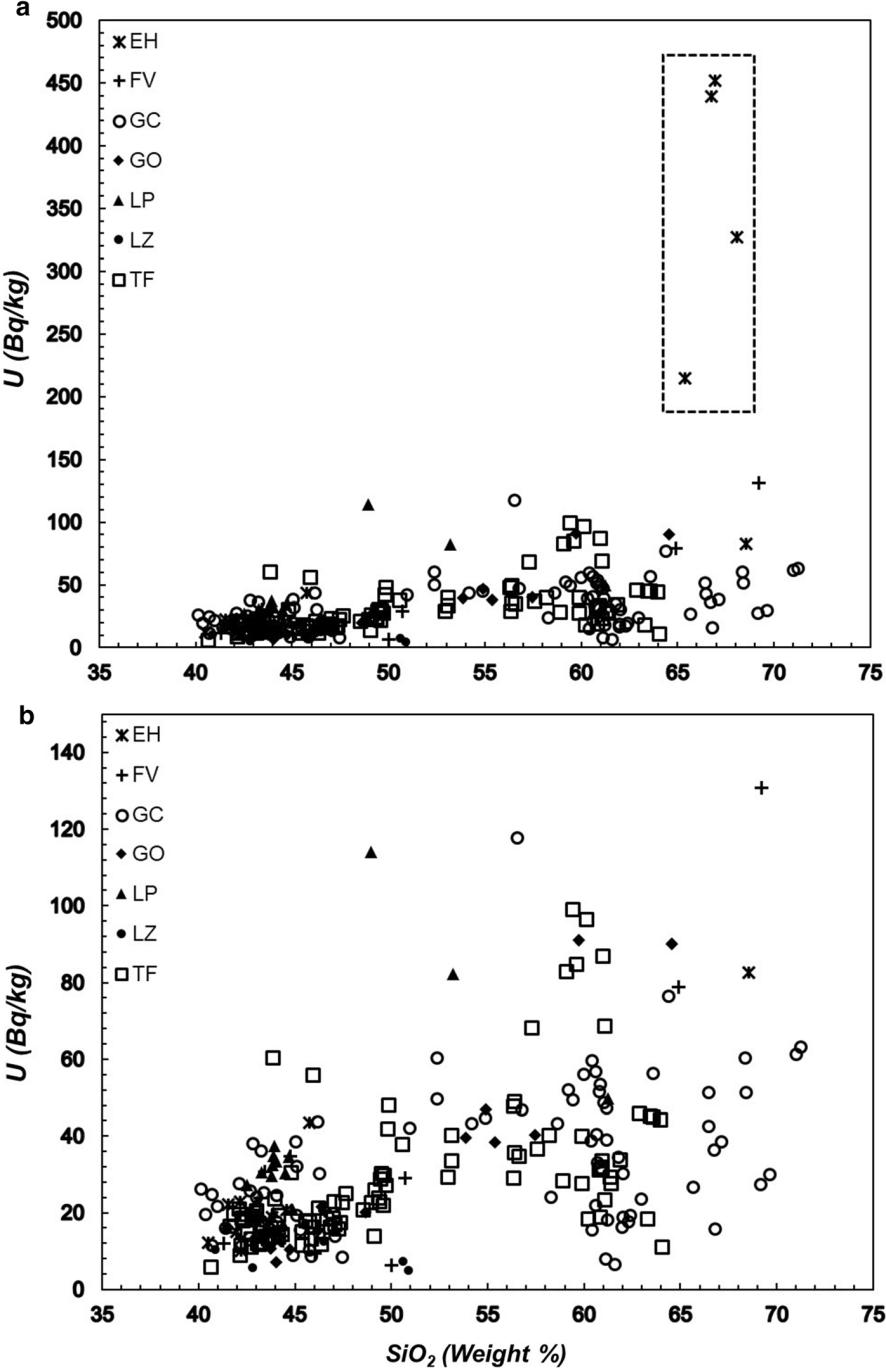
Specific activity of uranium versus silica content. Detection limits: SiO_2_ (0.01%), Th (0.12 Bq/kg); **a** included rhyolites from 2011 El Hierro eruption (Inside the rectangle), **b** after removal of rhyolites from El Hierro eruption. Symbols explanation: El Hierro (EH); Fuerteventura (FV); Gran Canaria (GC); La Gomera (GO); La Palma (LP); Lanzarote (LZ); Tenerife (TF). See text for details

**Fig. 7:**
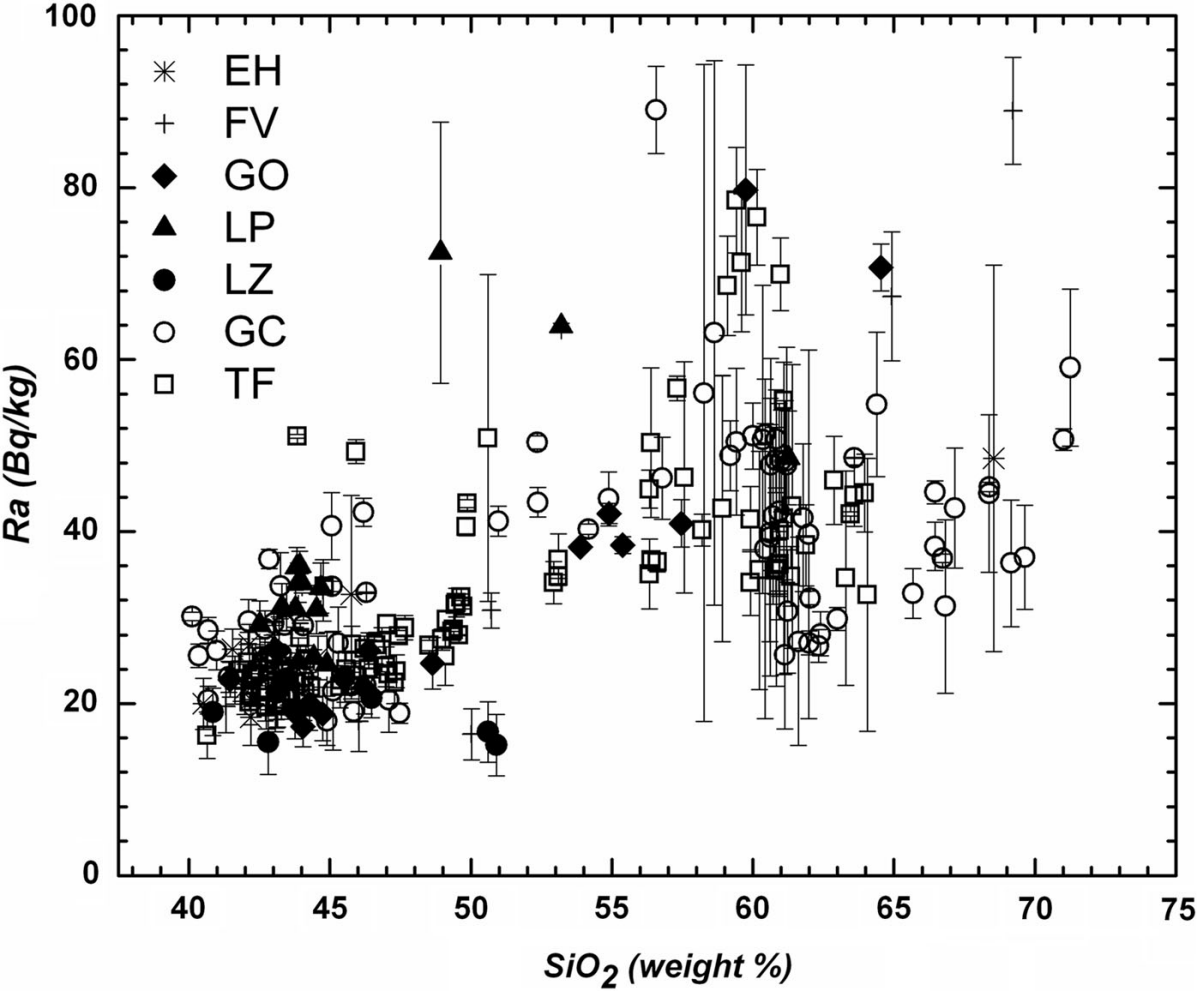
Estimated activity of radium versus silica content. Symbols explanation: El Hierro (EH); Fuerteventura (FV); Gran Canaria (GC); La Gomera (GO); La Palma (LP); Lanzarote (LZ); Tenerife (TF)

The distribution of the silica content (where the increase in silica content is indicative of a more magmatic differentiation) as a function of the activity of ^40^K (Fig. 4), ^232^Th (Fig. 5) and ^238^U (Fig. 6a, b) indicates that the lowest values of these radioactive elements are found in the low silica (mafic) samples. At larger silica content (more felsic samples), the specific activity of ^40^K increases, whereas the thorium and uranium activities exhibit a significant dispersion that needs to be explained with some detail. In the case of ^40^K vs silica content seems to be clear that this element content increases as silica content increases towards the most felsic samples but from silica content at approximately 60% the scatter of data increases. In the case of ^232^Th vs silica and ^238^U vs silica, the scatter of data exhibits a different path. As can be seen, the thorium concentration (Fig. 5) increases as silica content increases. The increase reaches extreme values when silica approaches to 60%. From this content of silica, the thorium concentration begins to decline until silica content reaches 70%. Figure 6 shows the relationship between uranium and silica. Figure 6a exhibits 4 points with anomalous concentrations on U (well above 200 Bq/kg). These four samples correspond to a rhyolitic material embedded on the inside of basanitic fragments erupted during the first 4 days of the El Hierro eruption in October 2011. The elevated content of U in the rhyolitic material is not reflected in the concentration of Th since the latter falls within typical values for Canarian rocks. In this regard, the U/Th ratio for these 4 samples is significantly larger than for the rest of the volcanic samples studied in this work. As such 4 anomalous concentrations of U are related to enrichments by secondary hydrothermal processes (Rodriguez-Losada et al., 2015), such anomalies were removed from the plot to a more appropriate interpretation of Fig. 6b. Here, the behavior of U vs silica seems to be like that of thorium. It can be noted an increasing rate from low silica content to values of 57–60% where content reaches its highest values and then marks a dominant downward trend to the highest silica contents (around 70%), as previously mentioned, a behavior similar to the one shown by thorium. A similar trend was found for the estimations of ^226^Ra (Fig. 7), namely low values at lower silica concentrations and higher and more disperse values at larger silica contents, whereas the specific activity of radium tends to decrease for samples with the largest silica concentrations.

The distribution of U, Th and Ra is common and also known in other trace elements such as Ba and Sr, where the initial increment of concentrations with increasing differentiation as silica content increases, is followed by a decrease in the most felsic members (Berlin & Henderson, 1969). Felsic rocks (mostly phonolites and trachytes in the Canaries) contain significant amounts of tectosilicates (mainly alkali feldspars and more rarely some quartz). The inclusion of these trace elements will continue in advanced stages of differentiation. Most of them are accommodated within the network of alkaline feldspars so that, with the massive crystallization of these, a greater amount of these trace elements will be included into the feldspars network. As at extreme stages of differentiation the components that make up feldspars are becoming scarce, feldspars crystallization declines and consequently, the residual liquid that will give rise to the most felsic or differentiated members will end up becoming depleted on the radioactive and trace elements as can be seen in the thorium–silica, uranium–silica and radium–silica relationships.

**Fig. 8:**
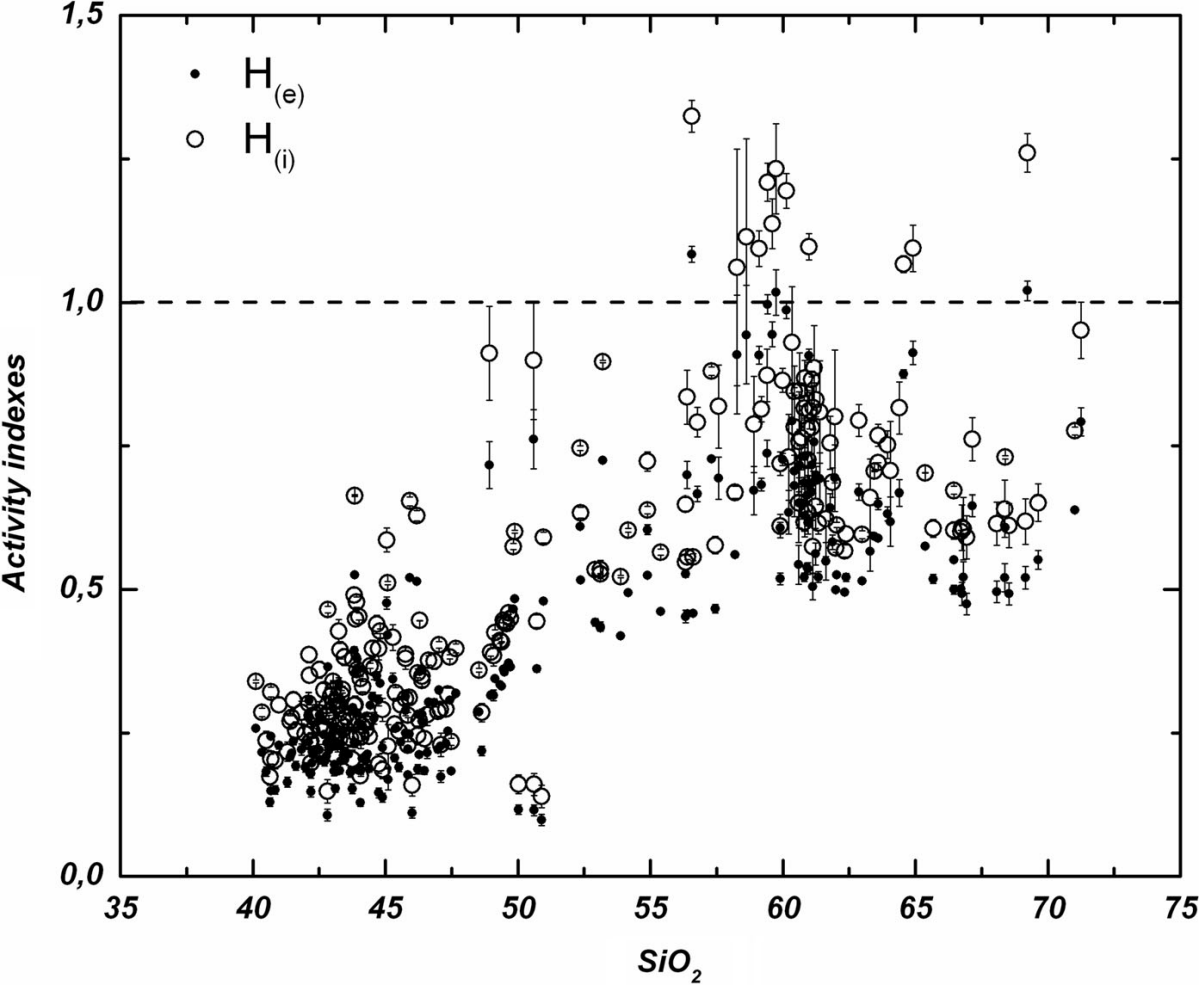
Geochemical variation diagram as a function of the external (H_(*e*)_) and internal (H_(i)_) hazard indexes. Dashed lines indicate the upper safety limit of 1 for both activity indexes

The combination of U, Th, K and Ra specific activities in terms of the hazard indexes of the rock samples (Fig. 8) closely follows their corresponding rates of magmatic differentiation. Both H_(e)_ and H_(i)_ are low in mafic rocks (more basaltic) and trend strongly upward in the felsic ones, exceeding in some cases the safety criterion. The scatter of the data is also larger at the felsic end of the sample distribution.

**Fig. 9:**
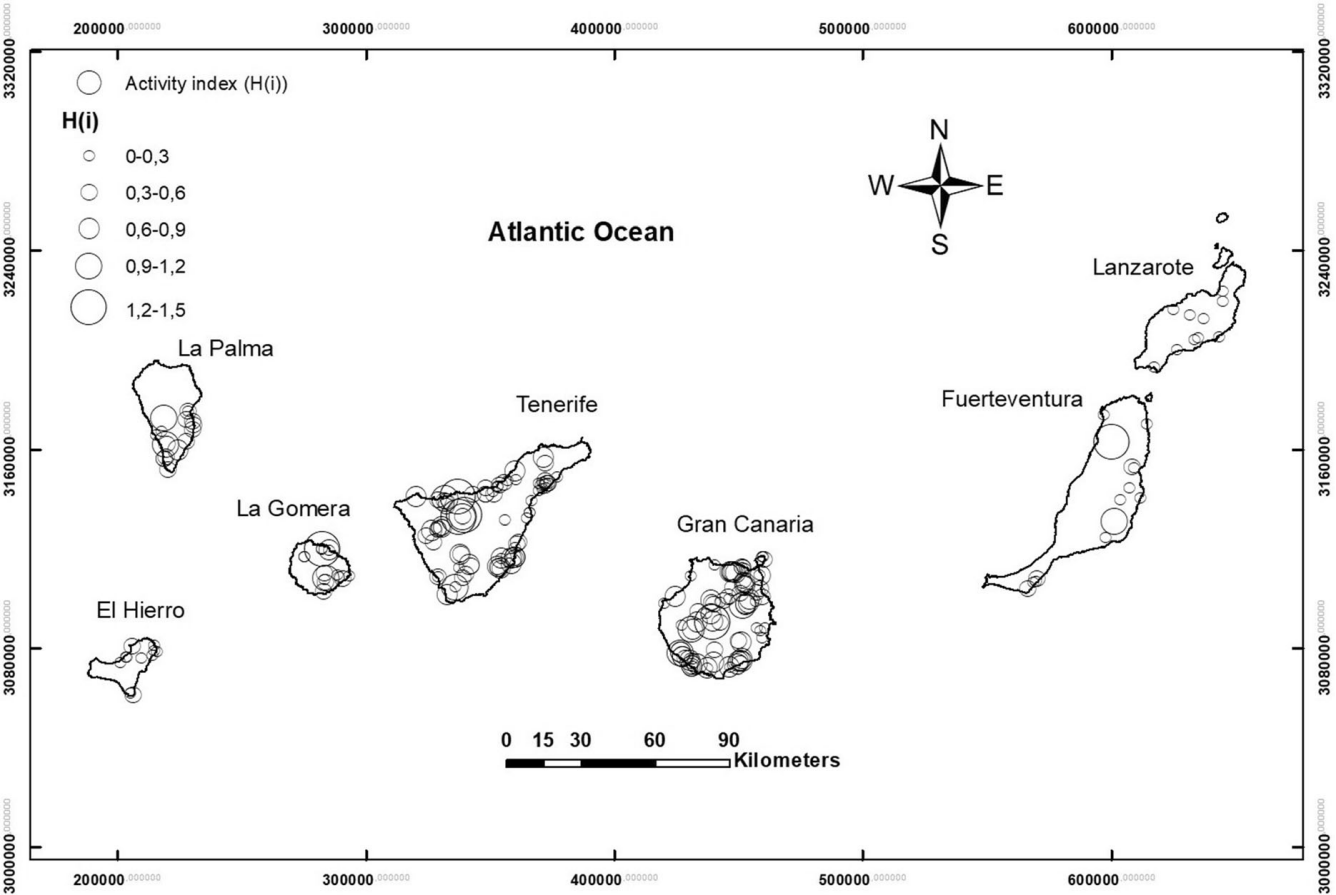
Map distribution of internal activity index (H_(i)_) in the province of Santa Cruz de Tenerife. The diameter of the symbols is a function of the internal activity index (H_(i)_). UTM coordinate system (wgs84)

The geographical distribution of the internal activity index in the Canary Islands (Fig. 9) is complex. In the islands of El Hierro and Lanzarote, the activity indexes are low and distributed homogeneously. This may be partly due to the basaltic nature of the volcanic formations on these islands and partly due to the sample selection effect. For the rest of the islands, it is possible to highlight some samples with particularly high values, located in areas of felsic formations, such as Cañadas and Teide-Pico Viejo edifices, in central and northern Tenerife. Elevated values of the internal activity index were also found in the felsic dome of Tindaya (northern of Fuerteventura Island), the trachytic-phonolithic subvolcanic formations in central Gran Canaria (Tejeda Caldera) or the trachytic-phonolithic complex in northern La Gomera.

**Fig. 10:**
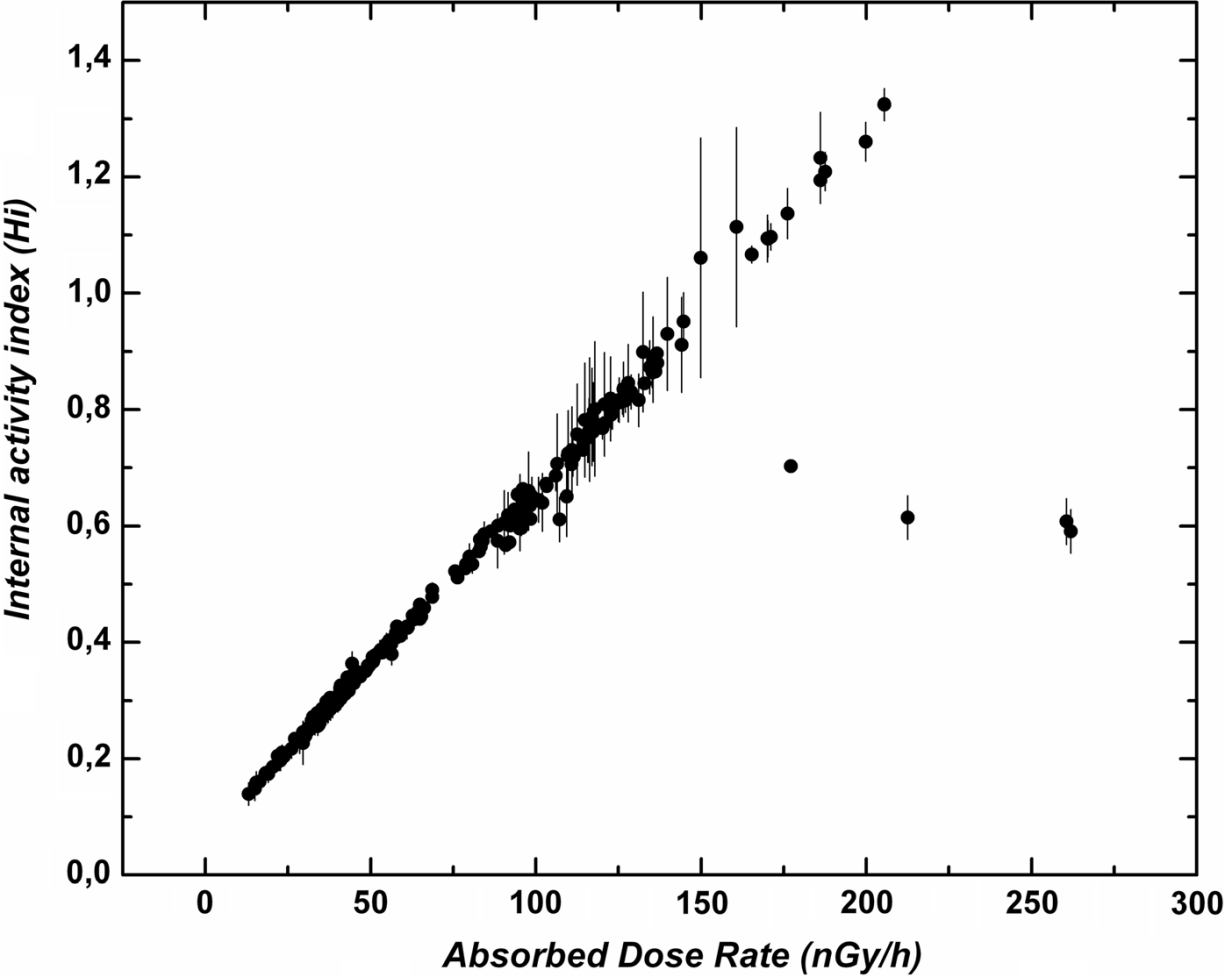
Estimated internal activity index as a function of the net dose rate for the studied samples of the Canary Islands

It is interesting to observe the distribution of *H*_(*i*)_ as a function of the absorbed dose rate in air (Fig. 10). As expected, there is a clear linear dependence between them since they both represent similar and interdependent parameters. However, there are some samples that do not follow the linear fitting, corresponding to the rhyolitic material included inside the fragments erupted during the first 4 days of the El Hierro eruption in October 2011 (see Fig. 6a). The uranium content of these samples is not only abnormally high due to secondary uranium enrichment (Rodriguez-Losada et al., 2015) but it is also in clear discordance with the thorium content. This highlights the existence of secondary enrichment in certain elements, such as uranium, that does not come from magmatic processes.

**Fig. 11:**
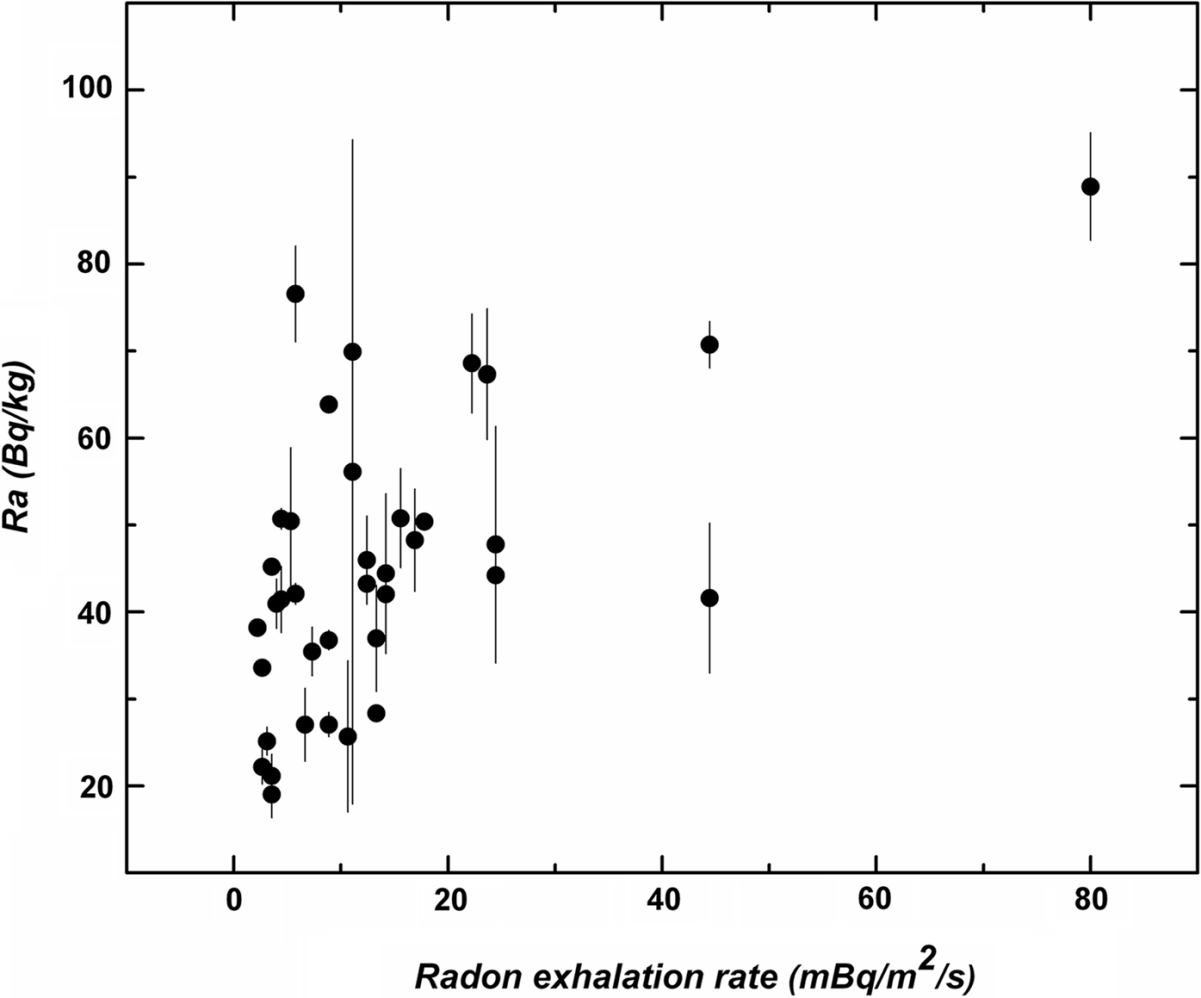
Radon exhalation rate (mBq/m^2^/s) vs activity of radium for a set of 37 selected samples of Canarian volcanic rocks

The radiological analysis of the Canarian data also contains the radon exhalation rates of the volcanic rocks. At present, the radon exhalation rate has been determined in a sample of 37 selected rocks covering a wide lithological spectrum (Fig. 11). The figure seems to indicate, despite the high dispersion, that there is an increase of the radon exhalation rate as the activity of radium increases. This is a promising result since there might be a statistically significant relation between the measured activity index and the potential concentration of radon in air.

## Radon-in-air activity at boreholes and wells

**Fig. 12:**
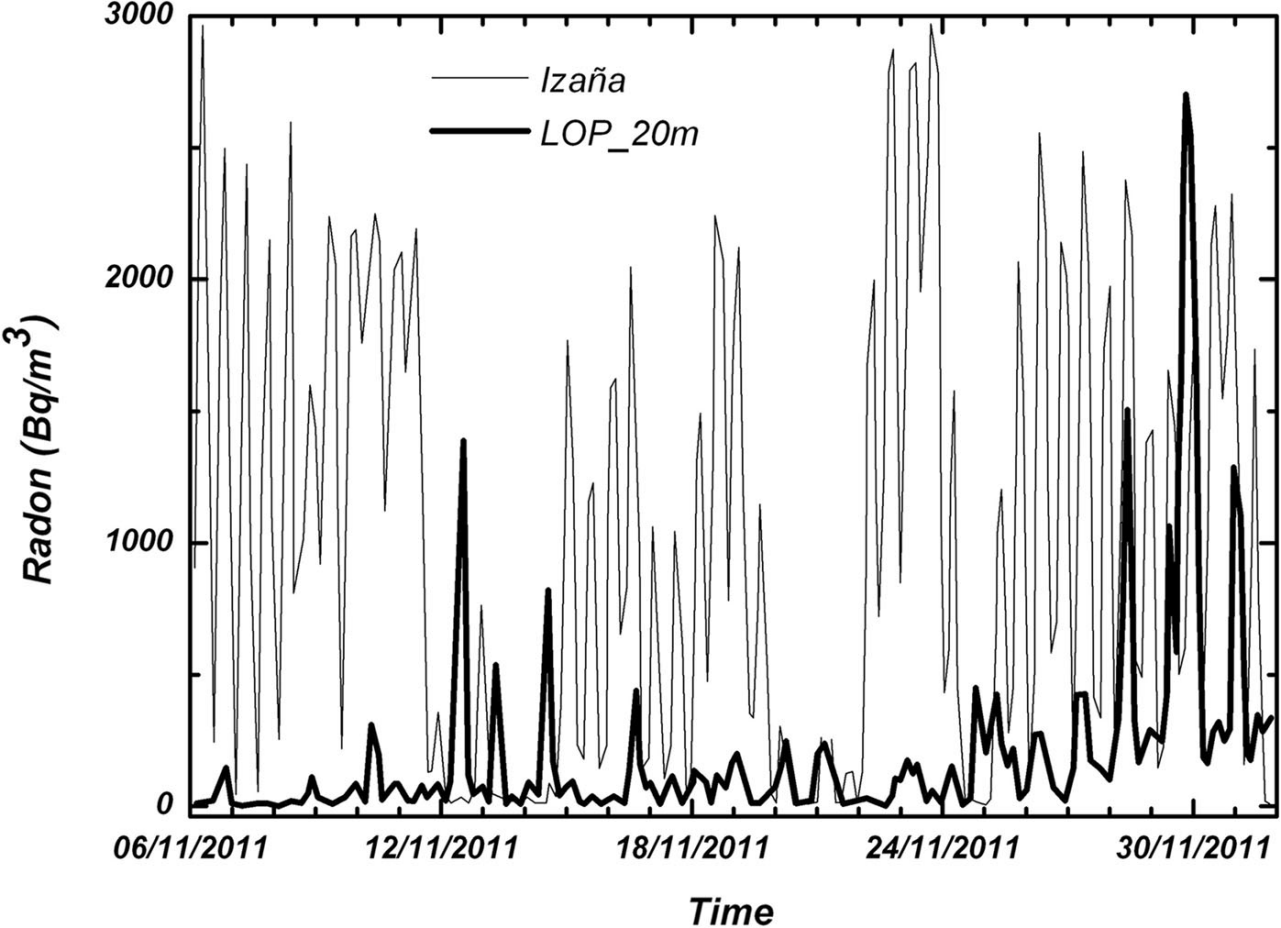
Radon concentration as a function of time for two of the boreholes drilled in Tenerife to study the transport of radon under different geological settings (thick line: Izaña Observatory, thin line: LOP_20m). See Table 2 for details

In previous works (i.e., Eff-Darwich et al., 2002), the temporal variations in the concentration of radon at subsurface levels were found to be modulated by atmospheric variables (air temperature and/or pressure) and the geological setting. This complex behavior was also found in the measurements that were carried out in the four boreholes and two wells that have been studied in this work, as illustrated in Fig. 12 and the following.

**Fig. 13:**
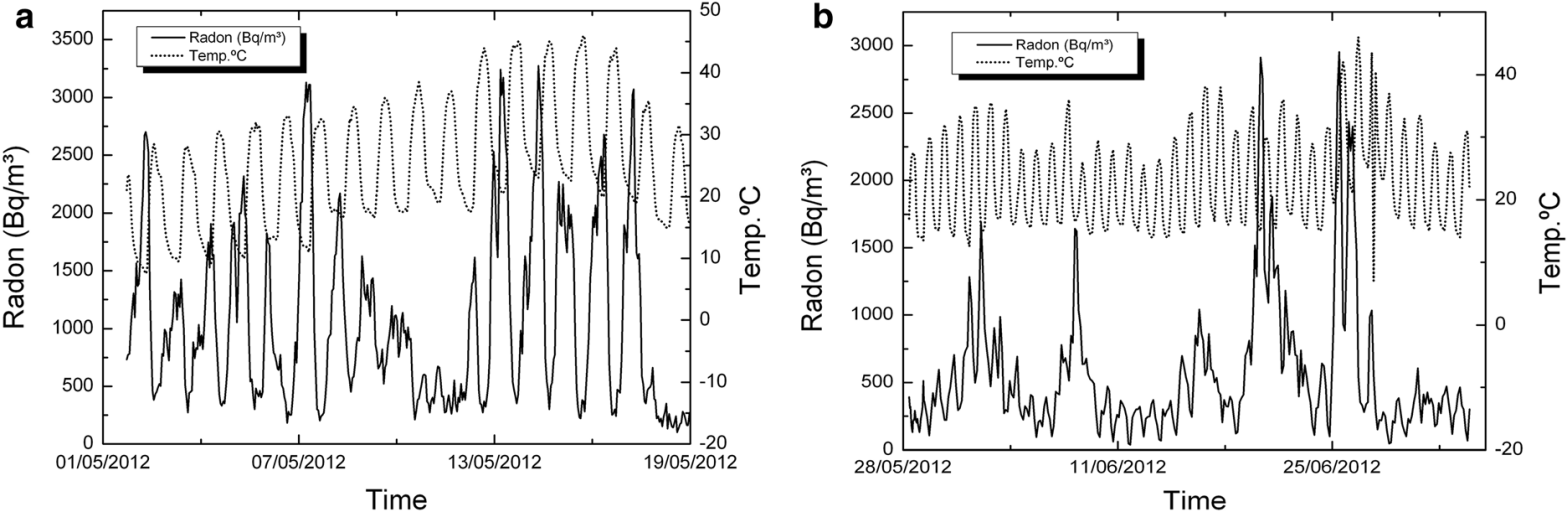
Radon-in-air activity and temperatures in the 1 m deep borehole from 1 to 19 May 2012 (**a**) and from 28 May to 4 July 2012 (**b**)

**Fig. 14:**
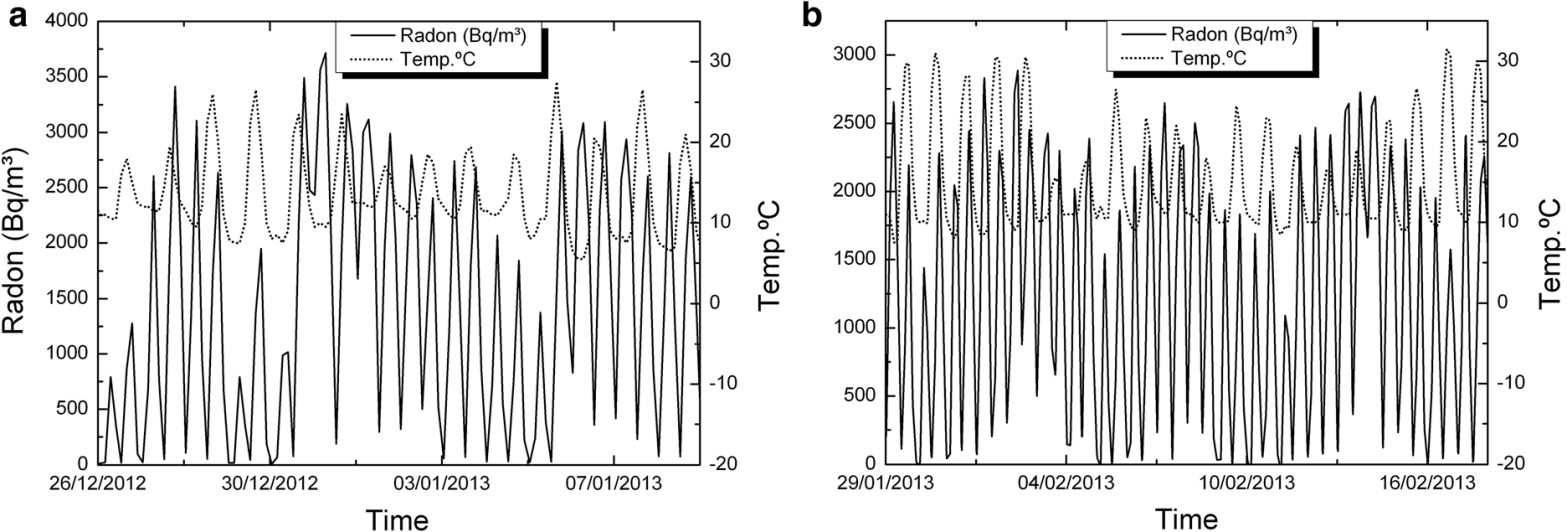
Radon-in-air activity and temperatures in the 20 m depth borehole from December 26 to January 9, 2012 (**a**) and from January 29 to February 18, 2013 (**b**)

**Fig. 15:**
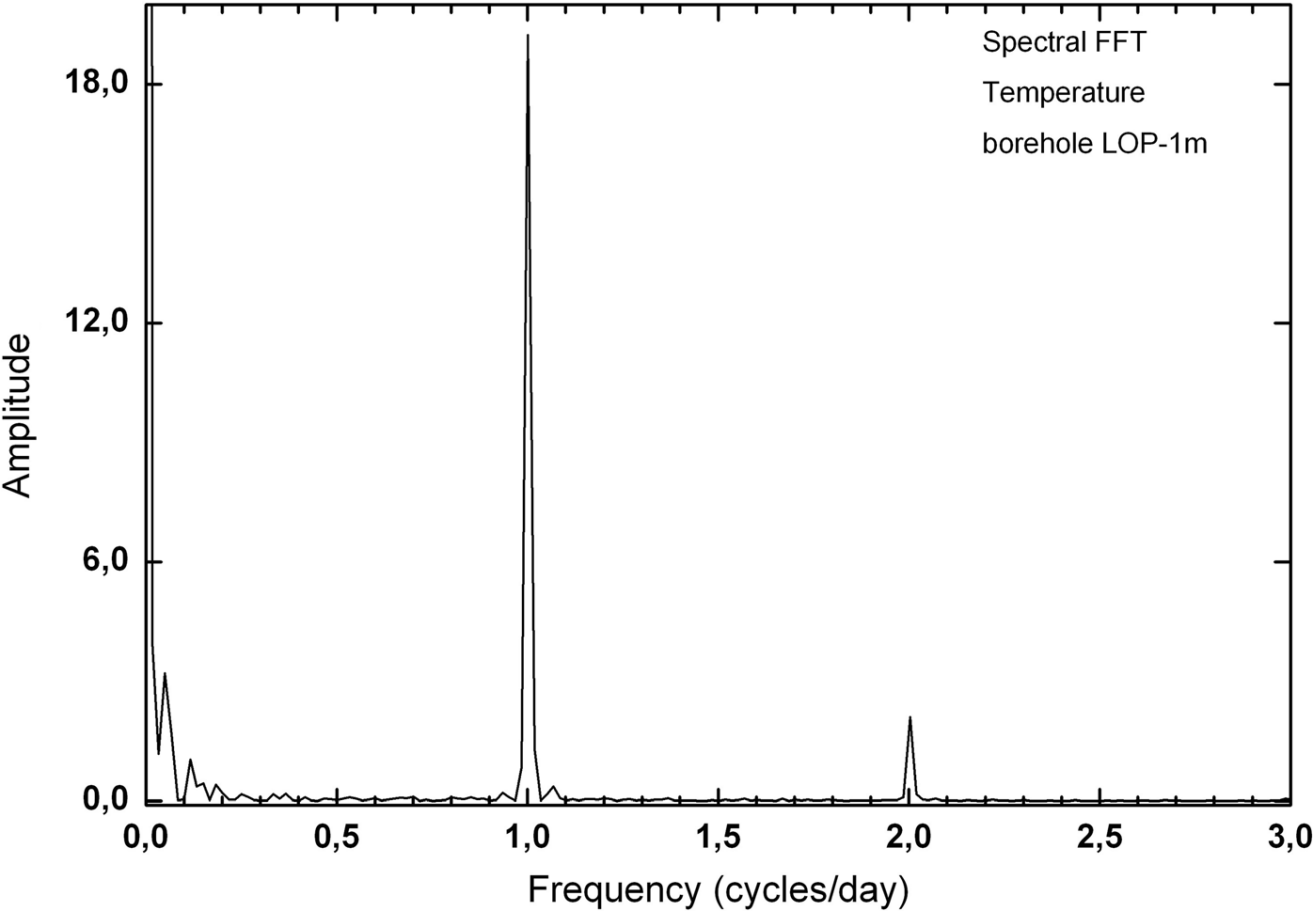
Spectral analysis of temperature variation in the lop-1 m borehole from September to November 2012

Measurements of radon activity in LOP_1m and LOP_20m boreholes were taken during a period that extends from May 2012 to February 2013 with data collection at intervals of 3 h (Figs. 13, 14, 15).

**Fig. 16:**
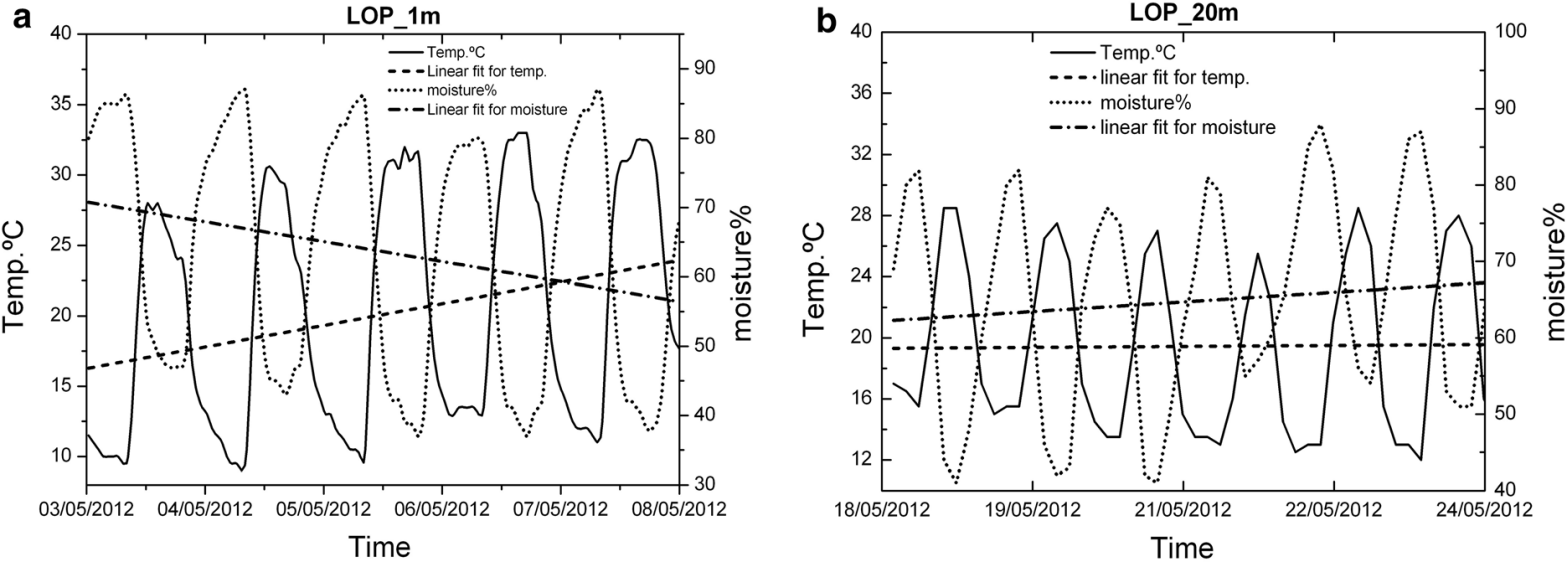
Typical detailed moisture and temperature variations for periods from 5 to 7 days in boreholes LOP_1m and LOP_20m

In both deep and shallow boreholes, it has been possible to observe the diurnal oscillations of temperature and moisture together with the diurnal cycles of radon activity. The most significant short period oscillation in the boreholes is 24 h, visible for temperature and humidity. In them, the variation pattern of both parameters can be clearly observed with a phase shift of 180° between them (Fig. 16). In the short-medium term, it is possible to identify other oscillation periods of temperature such as the one of almost weekly variation visible in the spectral analysis for temperature oscillation (Fig. 15) carried out with data from the 1 m depth borehole over a period of 2 months (peak near 0.15 cycles/day).

**Fig. 17:**
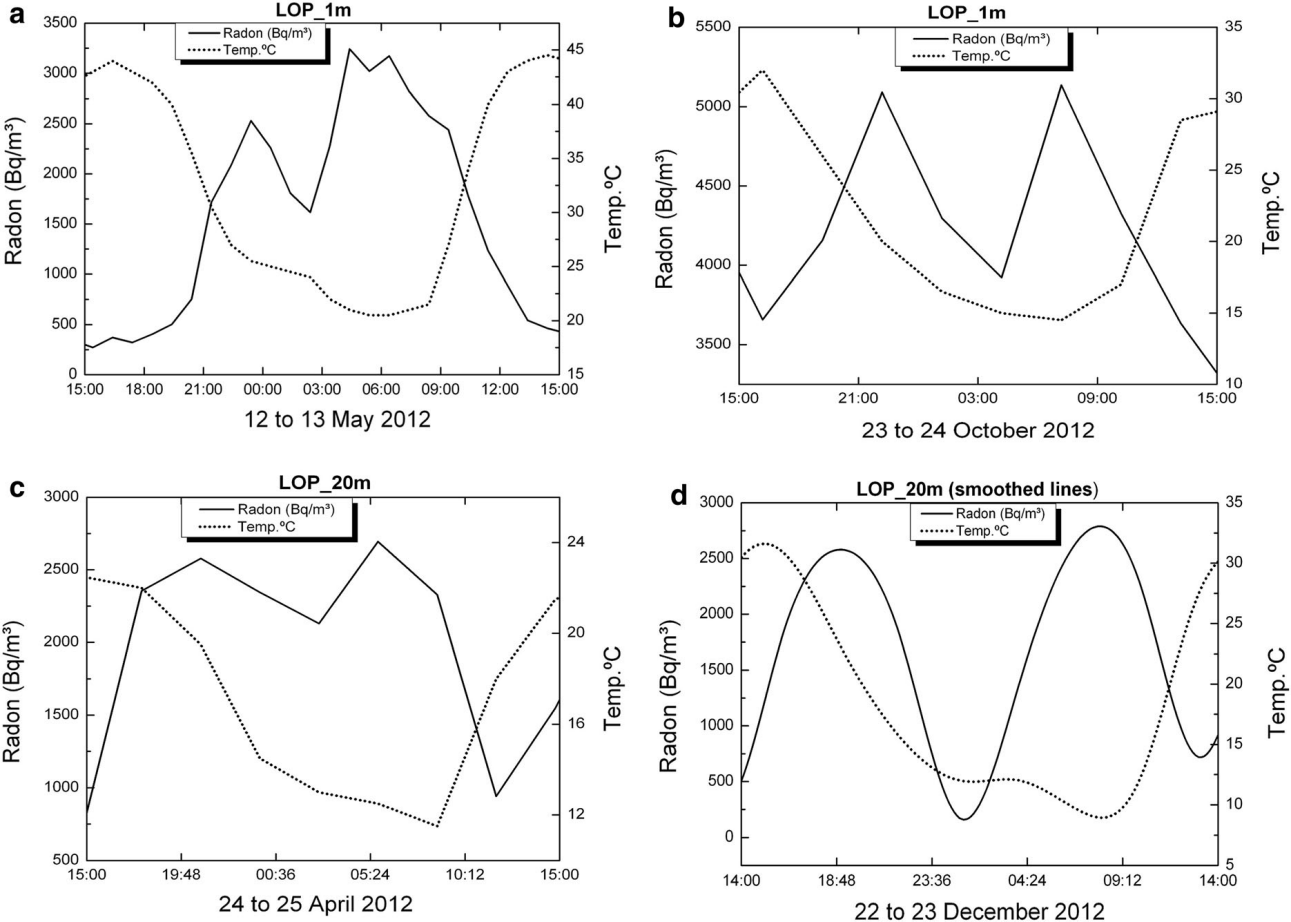
Variation of radon activity and temperature for a period of 24 h at the 1 m borehole (**a**, **b**) and at the 20 m borehole (**c**, **d**). See text for complementary explanation

Temporal variations in radon concentration are more complex that those found for temperature and humidity. In some cases, radon might exhibit two peaks of maxima per day and it goes in anti-phase relative to temperature variations (Fig. 17). In other cases, this behavior is not found.

**Fig. 18:**
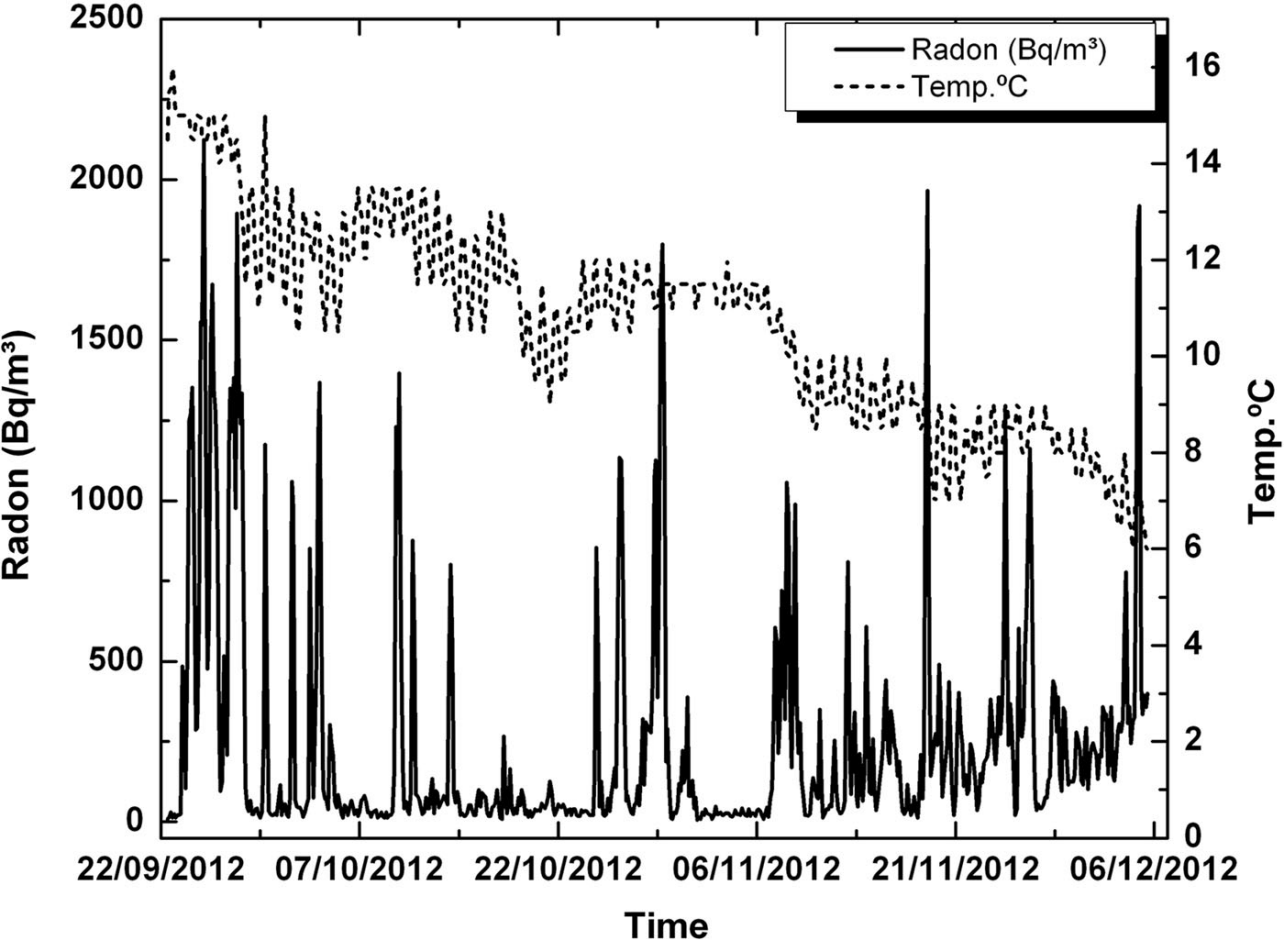
Radon and temperature at El Portillo well from 2012 September 22 to 2012 December 6

The radon sensor at El Portillo borehole was hanging just 1 m below the surface (Fig. 18). There is not clear relationship between radon and temperature variations, although it seems that background radon concentration started to increase as air temperature dropped below 8 °C, starting in December 2012.

**Fig. 19:**
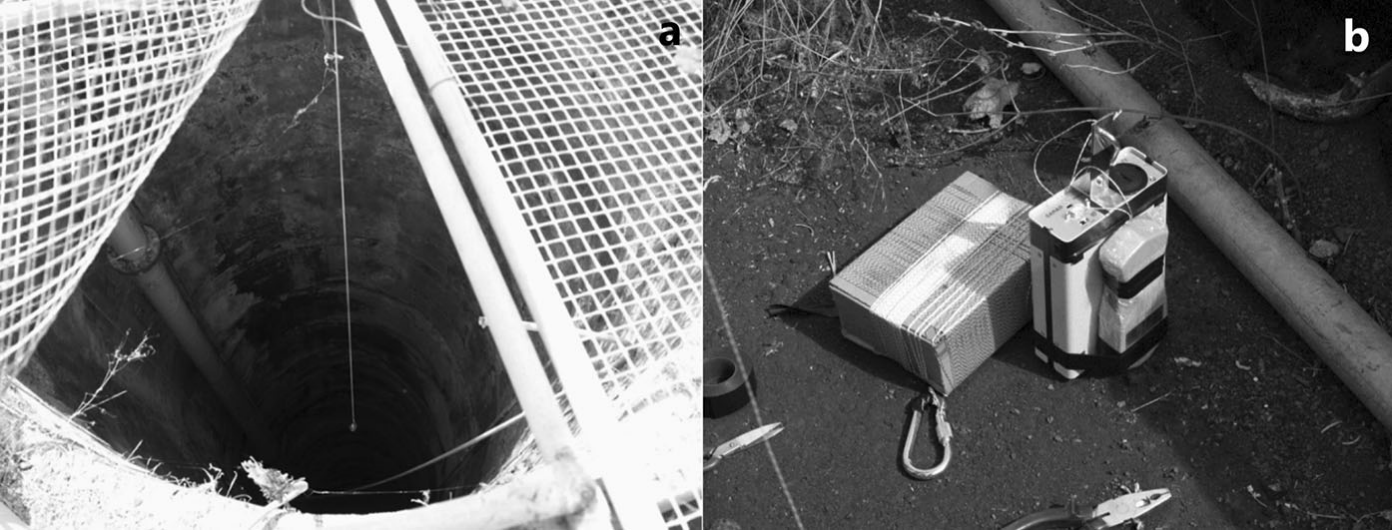
Startup of the measuring instruments for continuous radon-in-air monitoring in wells. View of the Los Llanillos well (**a**) and measuring instruments (**b**)

Two wells were selected to securely install the measurement equipment: Llanillos and Tejeguate (Fig. 19), both located at the El Golfo, in the El Hierro island, at an altitude of around 200 m. amsl (Table 2). In both wells, the instruments were collecting data (radon concentration, temperature and humidity) for about 1 year and 8 months, being only interrupted during the periodic battery change. It is thus possible to study long-term variations on radon activity.

**Fig. 20:**
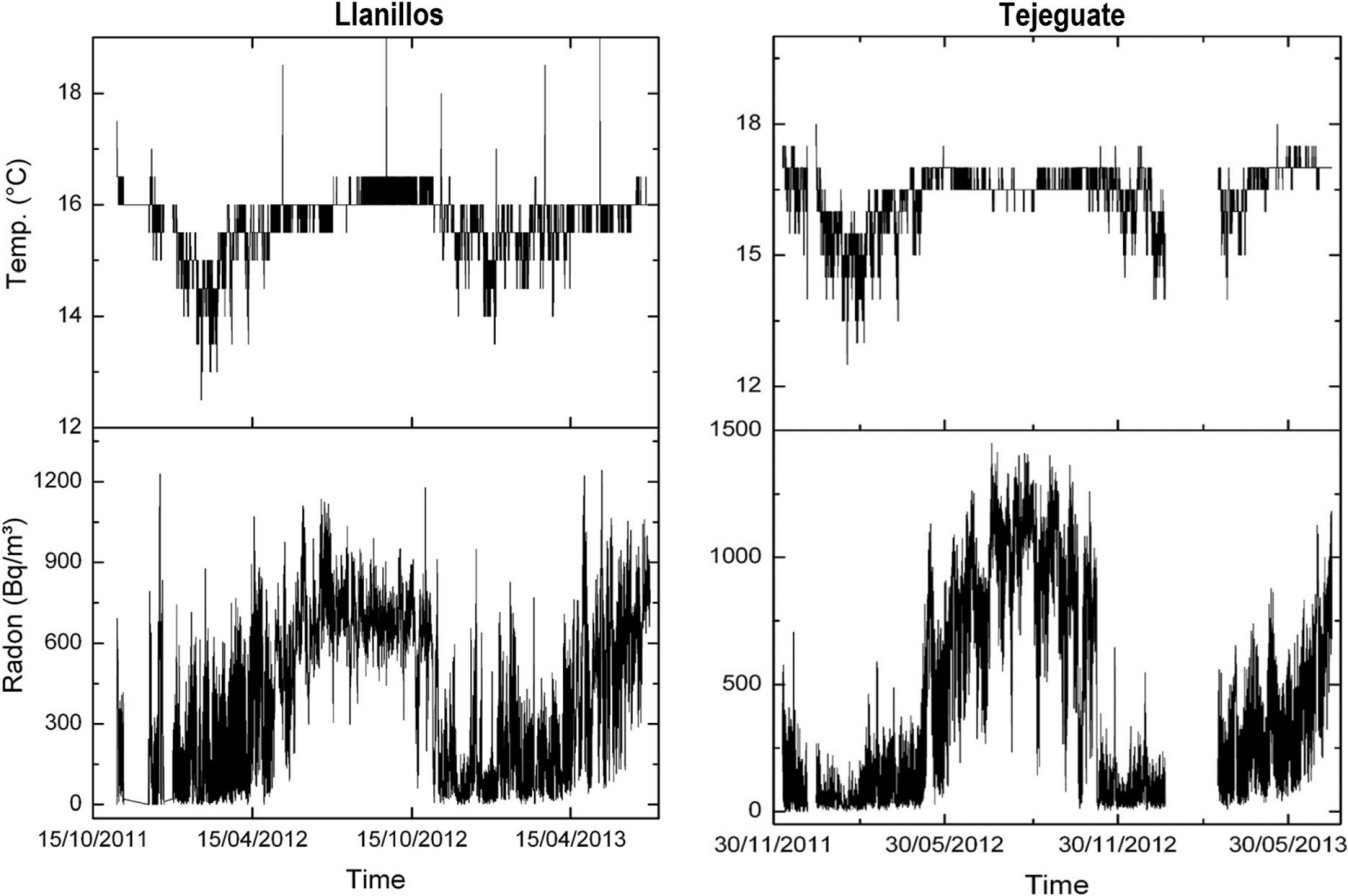
Long-term variation of radon activity and temperature at Llanillos and Tejeguate wells (El Hierro Island)

Radon concentration in both wells clearly shows a yearly pattern (Fig. 20), in which the activity of radon goes from a minimum of approximately 100 Bq/m^3^ to a maximum ranging from 750 to 1000 Bq/m^3^ (depending on the well). This temporal behavior seems to be correlated to the thermal regime within the wells.

**Fig. 21:**
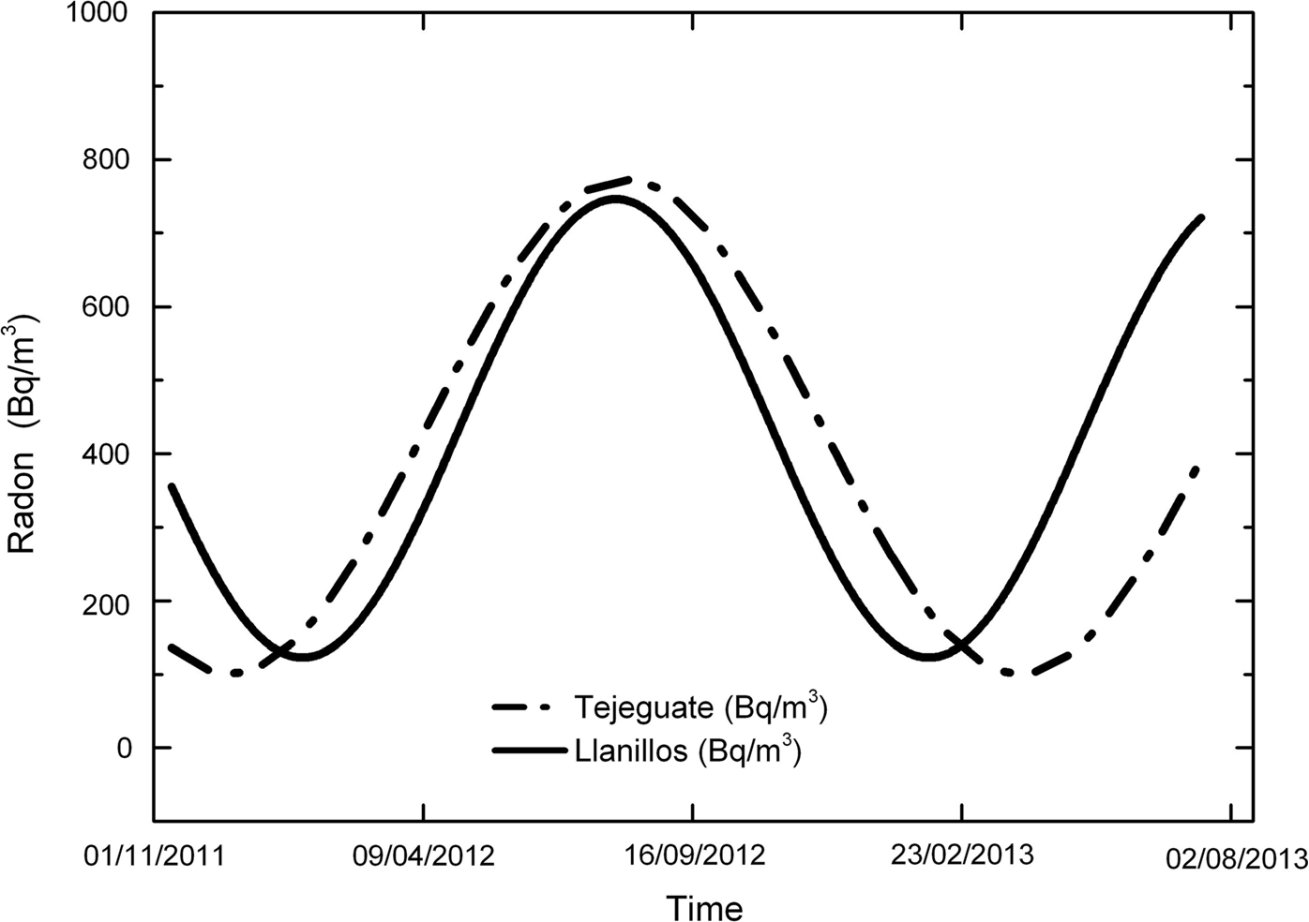
Comparison of radon activity of Llanillos and Tejeguate wells (El Hierro Island)

If a sinusoidal function is fitted to the variation in the radon concentration in both wells (Fig. 21), it can be seen there is a small mismatch between both fittings. Although part of the differences might be the result of instrumental adjustment, it cannot be ruled out that the different thermal response of each well is reflected in the release and transport of radon.

**Fig. 22:**
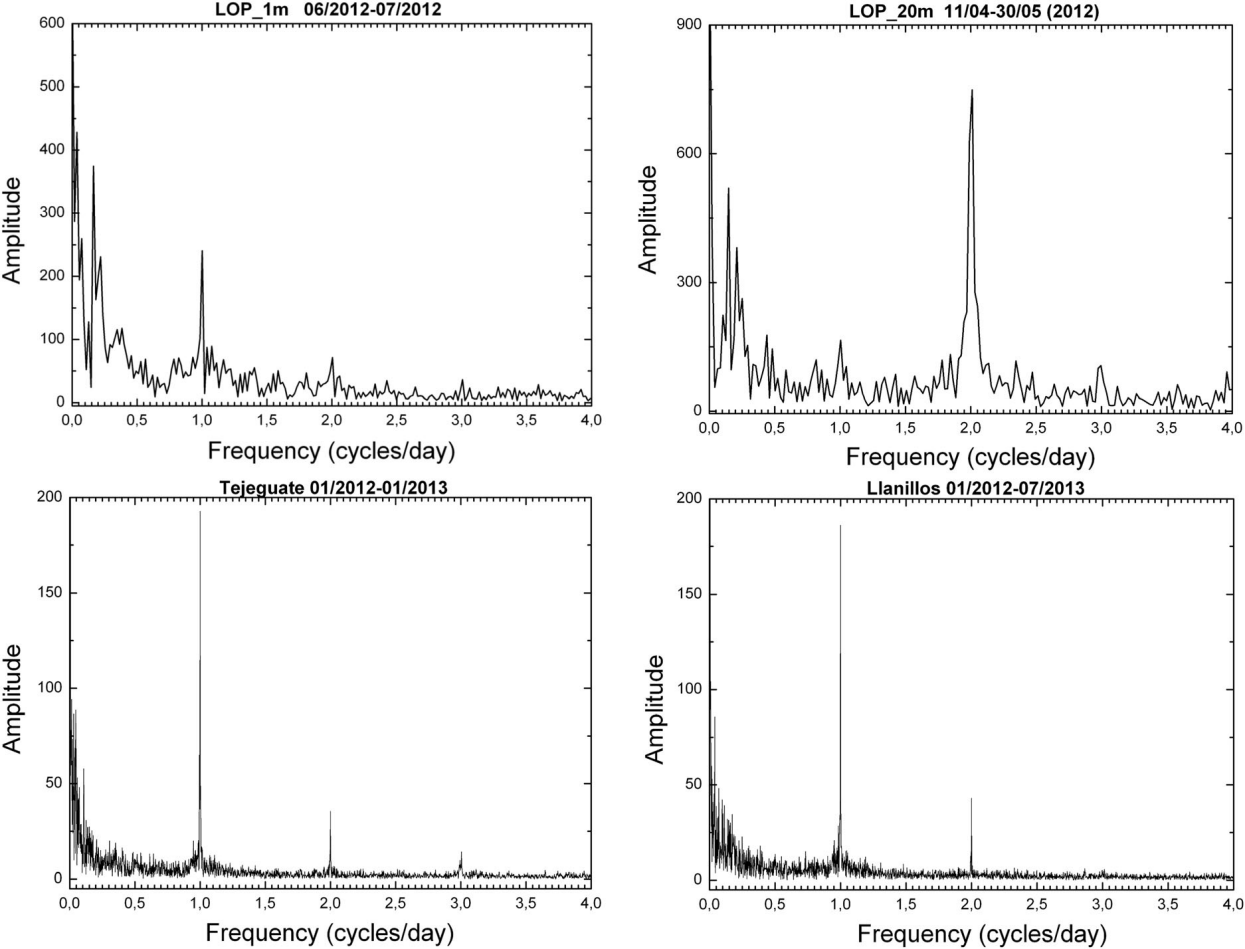
Spectral analysis of radon activity over time periods from one month at the LOP_1m and the LOP_20m boreholes (above) to several months at the wells of Tejeguate and Llanillos (below)

A more detailed study of the oscillations or cycles derives from the spectral analysis of the data about radon activity over time periods that vary from one to several months (Fig. 22). In this analysis, the oscillations of diurnal and semidiurnal periodicities stand out (frequencies of 1 and 2), being the one of semidiurnal more highlighted in the borehole of 20 m deep (LOP_20m) contrary to the one of 1 m deep (LOP_1m) in which the frequency of 1 cycle stands out more than 2 cycles per day. The wells of Llanillos and Tejeguate have similar spectrograms with respect to the oscillations of 1 and 2 daily cycles. It is also possible to differentiate oscillations of longer periods such as that of a 0.06 cycles/day (17 days) and almost weekly cycle, located approximately at a frequency of 0.12 cycles/day (8 days). Additionally, a 0.17 cycles/day (almost 6 days) appear clearly visible in both boreholes of 1 and 20 m depth).

## Discussion and conclusions

Here we have two types of research on radon concentration that have been carried out. First, the relationship between radon concentration and lithology, as well as the impact on risk levels, has been studied. In the basis of the specific activities of unstable elements such as uranium, thorium, radium and potassium, different activity indices were estimated for each sample, making it possible to estimate the risk levels of radioactive hazard depending on the lithology. The most significant aspect of this issue is that the activity indices exceed the safety threshold of 1 in more differentiated or felsic rocks, with silica content around 55% or higher (Fig. 8). Based on the sampling map (Fig. 1) and subsequent sample selection, a map was elaborated containing the values of the internal activity index for the collected samples in the Canary Islands (Fig. 9). It is remarkable that the area of Las Cañadas del Teide and the north coast of Tenerife, near the village of La Guancha, dominated by more differentiated rocks (trachytes and phonolites) exhibits high values of the internal activity index. It is also remarkable the area of Tindaya rhyolitic dome, to the north of Fuerteventura, predominantly dominated by felsic rocks and where the highest levels of activity in the entire Canary archipelago are reached. Likewise, it is remarkable the presence of samples with elevated internal activity index, all of them of trachytic–phonolitic type, related to the subvolcanic formations of the central sector of Gran Canaria (Tejeda Caldera) or to the trachytic–phonolithic complex of the northern part of La Gomera (Fig. 9). On the other hand, but related to the latter, it can be observed that the lowest values in the content of the radioactive elements, potassium, thorium, uranium and radium were found in the mafic (basaltic) samples, whereas the largest were in the felsic ones. However, for potassium, a slight decrease in potassium content can be perceived from a silica content over 60% in weight. Despite the greater degree of data scatter towards the felsic end, in the case of thorium, uranium and radium it can be noted an increasing rate from low silica content to values of 57 to 60% where the content reaches its highest values and then marks a dominant downward trend to the highest silica contents (around 70%). This behavior, revealed for other trace elements contained in volcanic rocks (Berlin & Henderson, 1969), is attributed to the massive crystallization of feldspars in advanced stages of magmatic differentiation, incorporating the greatest amount of these elements and leaving the residual liquid, which will give rise to the most felsic rocks, progressively depleted in them (K, Th, U and Ra).

A second goal is focused on the variations of radon-in-air activity inside boreholes and wells over different time intervals. The interest of this issue is that it has made it possible to carry out short- and long-term radon measurements, revealing a series of regular cycles in radon activity that vary from daily to weekly and annual cycles already widely documented in previous studies (Botha et al., 2018; Eff-Darwich et al., 2002; Groves-Kirkby et al., 2010; Li et al., 2006, among others). The influence that the geological setting can exert on radon activities was already revealed in previous works (Eff-Darwich et al., 2002; Gillmore et al., 2005). Here, it is possible to see how on different conditions and settings, very different radon activity records can be obtained from one location to another (Fig. 12). Moreover, long-term measures taken in two wells located on the island of El Hierro show a seasonal cycle variation in radon activity (Fig. 20), with a maximum of activity around August–September and a minimum, around February–March. This roughly matches with the annual thermal cycle at El Hierro, where the average maximum temperature coincides around September and the thermal minimum, around February. The increase in radon activity levels, higher during the summer season, has been reported in several previous works for home interiors and in soils (Bochicchio et al., 2005; Eff-Darwich et al., 2002; Moreno et al., 2016; Omori et al., 2009; Papaefthymiou et al., 2003; Singh et al., 2005), in which there is a pattern that matches with the one reported here in our monitorized wells.

Conversely, Botha et al., (2018), studying the radon-in-air activity at semidiurnal, diurnal and annual timescales found that winter radon levels were significantly higher than in summertime (peaks of maximum radon activity around the months of June and July), explained as due to an increased frequency of winds from the NNW, associated with continental air masses. In our case, the Canary Islands are located at the northern hemisphere, away from any continental influence except for short periods of time, in which the NE trade winds, are eventually replaced by winds from ESE, coming from the Sahara Desert, much drier and dusty (known as “calima”). This means that a mechanism like that explained by Botha et al. cannot be invoked in the case for this work. In addition, Cigolini et al., (2009), in their study about the influence of soil temperature, atmospheric pressure and tidal forces on radon degassing at Stromboli volcano, found a negative correlation between radon emissions and seasonal temperature variations, in a way, something like that described by Botha et al., (2018) and just the opposite results to those found in this work for the wells interior. However, while the measurements taken by Cigolini et al. were carried out on the same surface of the soil or a few centimeters inside it. In the case of this work, as already stated, the measurements took place at 50 m depth inside the wells, conditions that are not analogous to the previous case. Likewise, at the Stromboli volcano there is a significant nearby active volcanic system dominating the release mechanisms which does not occur in the area of the wells studied here. Consequently, release mechanisms like those of Stromboli volcanic system do not work in the case of the wells from El Hierro.

Seasonal variations like the ones found in this work and just already revealed in previous studies (Martin-Luis et al., 2002; Moreno et al., 2016; Omori et al., 2009) are explained as caused by atmospheric temperature and precipitation. Here, in an environment dominated by wet and fresh ocean winds from the NE (trade winds), it is suggested that a general warming of the atmospheric air near the ground, promote a seasonal relative depression at the surface during the summer season with the corresponding positive pressure anomaly at the well outlet due to a greater outflow of radon and other gases from the interior of the earth towards the surface while during winter time, a relative slight overpressure near the ground accompanied by the general decline or absence of the trade winds, may induce a dominant net inflow from the surface to the earth interior and the consequent depletion of the radon-in-air activity inside the wells. This kind of behavior was already exposed before for radon measurements in galleries on the island of Tenerife (Martin-Luis et al., 2002).

At short-term periods (daily or weekly), no correlation between radon activity and temperature was observed in the wells. The roughly annual coincidence between radon activity and temperature throughout the annual cycle in the wells contrasts with the dominant negative correlation between radon activity and temperature in the short-term measurements taken in the boreholes (diurnal cycle with negative correlations around −0.7 in the shallow borehole LOP_1m, while in the deep borehole (LOP_20m), the daily correlations may vary from positive (0.3) to a most frequent negative correlation (up to −0.4). For intervals greater than one day the correlation remains negative in the shallow borehole and tends to zero in the deep borehole, suggesting that for different daily intervals in the referred deep borehole, the correlation can be sometimes positive or negative depending on the selected daily interval. This means that the environmental parameters that control radon emission do not work in the same way in wells (measured at deeper levels) with respect to the boreholes (measured at shallow levels) as well as in the short-term with respect to the long-term measurements (Figs. 17, 20). Focusing on short periods cycles, the opposite behavior of radon activity and air temperature along a diurnal cycle shown in this work (Fig. 17) was previously reported in other studies (Omori et al., 2009). Omori et al. found that in a diurnal cycle, the highest values of radon in air concentrations are conditioned by minimum values of air temperature, wind speed and radiation balance and consequently found that maximum radon concentration occurs during nighttime coinciding with greater nighttime atmospheric stability. Other factors such as tidal forces seem to play a relevant role on the radon-in-air activity together with the environmental factors (Cigolini et al., 2009; Groves-Kirkby et al., 2006). The appearance of one and two daily peaks of radon concentration (Fig. 17), although not seen so clearly for successive days, seems to have its origin in gravitational variations due to the apparent turn of the moon and sun over 24 h around the Earth in accordance with the already defined diurnal and semidiurnal cycles due to tidal forces (Pugh, 1987). The cycle of approximately 17 days detected in some cases and perceptible in the attached spectrograms (peaks near 0.06 cycles/day in Fig. 22), may be caused due to the Luni-solar fortnightly tidal influence. Another almost weekly cycle is found in the spectrograms of the boreholes (peaks near 0.15 cycles/day in the LOP_1m and LOP_20m boreholes) where the measurements were carried out at shallow levels (less than a meter from the surface). This weekly cycle was not evident in the wells, where measurements were carried out at deeper levels (50 m below the surface) as can be deduced from the corresponding spectrograms (Tejeguate and Llanillos). This weekly cycle is induced by the weekly cycle of thermal variation detected in the spectral analysis of Fig. 15.

## Data Availability

Data sharing is not applicable to this article as no datasets available via web link were generated during the current study. Nevertheless, additional data may be provided from the authors after reasonable request. Code availability is not applicable in this section.
